# The Emerging Role of Nutritional Vitamin D in Secondary Hyperparathyroidism in CKD

**DOI:** 10.3390/nu10121890

**Published:** 2018-12-03

**Authors:** Chien-Lin Lu, Dong-Feng Yeih, Yi-Chou Hou, Guey-Mei Jow, Zong-Yu Li, Wen-Chih Liu, Cai-Mei Zheng, Yuh-Feng Lin, Jia-Fwu Shyu, Remy Chen, Chung-Yu Huang, Kuo-Cheng Lu

**Affiliations:** 1 Graduate Institute of Clinical Medicine, College of Medicine, Taipei Medical University, Taipei 11031, Taiwan; janlin0123@gmail.com (C.-L.L.); athletics910@gmail.com (Y.-C.H.); wayneliu55@gmail.com (W.-C.L.); 11044@s.tmu.edu.tw (C.-M.Z.); linyf@shh.org.tw (Y.-F.L.); shyujeff@mail.ndmctsgh.edu.tw (J.-F.S.); 2 Division of Nephrology, Department of Medicine, Fu Jen Catholic University Hospital, School of Medicine, Fu Jen Catholic University, New Taipei City 242, Taiwan; 039666@mail.fju.edu.tw (G.-M.J.); roy_lii@yahoo.com.tw (Z.-Y.L.); 3 Division of Cardiology, Department of Medicine, Fu Jen Catholic University Hospital, School of Medicine, Fu Jen Catholic University, New Taipei City 242, Taiwan; ocean0829@yahoo.com; 4 Division of Nephrology, Department of Medicine, Cardinal-Tien Hospital, School of Medicine, Fu Jen Catholic University, New Taipei City 23155, Taiwan; 5 Division of Nephrology, Department of Internal Medicine, Tungs’ Taichung MetroHarbor Hospital, Taichung City 433, Taiwan; 6 Division of Nephrology, Department of Internal Medicine, Shuang Ho Hospital, New Taipei City 235, Taiwan; 7 Department of Internal Medicine, School of Medicine, College of Medicine, Taipei Medical University, Taipei 11103, Taiwan; 8 Department of Biology and Anatomy, National Defense Medical Center, Taipei 114, Taiwan; 9 Kidney Dialysis Center, Kamifukuoka General Hospital, Saitama 356, Japan; remyneko@yahoo.co.jp; 10 Department of Medicine, Show-Chwan Memorial Hospital, Changhua 50008, Taiwan; c_y_huang_alex@yahoo.com.tw

**Keywords:** calcitriol, calcimimetics, nutritional vitamin D, secondary hyperparathyroidism

## Abstract

In chronic kidney disease (CKD), hyperphosphatemia induces fibroblast growth factor-23 (FGF-23) expression that disturbs renal 1,25-dihydroxy vitamin D (1,25D) synthesis; thereby increasing parathyroid hormone (PTH) production. FGF-23 acts on the parathyroid gland (PTG) to increase 1α-hydroxylase activity and results in increase intra-gland 1,25D production that attenuates PTH secretion efficiently if sufficient 25D are available. Interesting, calcimimetics can further increase PTG 1α-hydroxylase activity that emphasizes the demand for nutritional vitamin D (NVD) under high PTH status. In addition, the changes in hydroxylase enzyme activity highlight the greater parathyroid 25-hydroxyvitmain D (25D) requirement in secondary hyperparathyroidism (SHPT); the higher proportion of oxyphil cells as hyperplastic parathyroid progression; lower cytosolic vitamin D binding protein (DBP) content in the oxyphil cell; and calcitriol promote vitamin D degradation are all possible reasons supports nutritional vitamin D (NVD; e.g., Cholecalciferol) supplement is crucial in SHPT. Clinically, NVD can effectively restore serum 25D concentration and prevent the further increase in PTH level. Therefore, NVD might have the benefit of alleviating the development of SHPT in early CKD and further lowering PTH in moderate to severe SHPT in dialysis patients.

## 1. Introduction

Secondary hyperparathyroidism (SHPT) is the most common complication of advanced renal disease, and an independent risk for all-cause mortality and cardiovascular mortality. The prevalence of SHPT within chronic kidney disease (CKD) population is highly variable, and dependent on the definition of SHPT, renal disease severity, phosphorus burden and vitamin D status. The prevalence of SHPT in patients with CKD stage 4 was estimated at 77.9% (PTH > 70 pg/mL) and 54.1% (PTH > 110 pg/mL) in Spain [1] and within the dialysis population in Europe was ranged from 30–49%, Americas (US, Canada) 54% and Japan 11.5% [2]. The Kidney Disease Improving Global Outcomes (K-DIGO) CKD-MBD Guideline in 2017 suggested that calcitriol or other vitamin D receptor activator (VDRA) analogs should not be routinely used in CKD stages 3–5 patients due to the risk of vascular calcification [3]. A markedly increased parathyroid hormone (PTH) level prompts the initiation of PTH lowering therapy in advanced CKD patients. Either calcitriol, calcimimetics, or combination therapy is the choice of treatment in SHPT CKD patients. Nevertheless, 20–30% of SHPT patients are treatment resistant to nonselective VDRA analogs such as calcitriol, whose serum PTH does not decrease, even with an increased dose of VDRA analogs [4]. Neither new generation VDRA analogs or calcimimetics can attenuate such treatment resistance [5,6].

Vitamin D deficiency (VDD) is a global public health problem in the general population, with prevalence ranging from 20% and 100% [7,8,9]. The elderly, areas of higher latitudes, people with darker skin tone, men and people who are overweight or obese individuals are all at high risk for VDD [10]. In addition, several studies have also demonstrated that CKD is a significant risk factor of VDD [11,12,13]. Gonzalez et al. reported that most hemodialysis (HD) patients (97%) had low 25D levels [12]. Our previous studies also showed a high prevalence of low vitamin D levels in HD patients with SHPT [14,15,16,17,18].

Recent studies have highlighted the benefits of nutritional vitamin D (NVD) supplement in areas outside of traditional bone and mineral metabolism in the general population. Several observation studies and randomized controlled trials have demonstrated that NVD replacement improved the bone remodeling markers [19,20,21], but whether such improvements translate into being clinically significant in CKD and SHPT is not yet clear. This article reviewed the pathophysiology of SHPT and emerging role of NVD replacement in SHPT prevention in early CKD patients and the additive role of NVD in furthering the PTH lowering effect in moderate to severe SHPT in dialysis patients.

## 2. The Pathophysiology of Secondary Hyperparathyroidism

As the glomerular filtration rate decreases in CKD progression, phosphate begins to accumulate due to the decrease in the functional nephron number. In addition, 1,25-dihydroxy vitamin D (1,25D) produced in the remaining kidney is decreased and renal 1α-hydroxylase activity is further inhibited by fibroblast growth factor-23 (FGF-23) and other uremic factors that lead to 1,25D deficiency. Both phosphate burden and 1,25D deficiency cause hypocalcemia and stimulate PTH secretion from PTG, called SHPT. The PTH synthesis, transcription, and parathyroid cell proliferation are mainly regulated through serum calcium and 1,25D level. Both hypocalcemia and 1,25D deficiency among CKD patients result in PTH secretion and PTG hyperplasia [22] and consequently result in unbalanced bone remodeling, soft tissue/vascular calcification, and increases the risk of cardiovascular event and all-cause mortality [23,24,25,26]. Recently, evidence has emerged supporting the role of FGF-23 as the primary event in the pathogenesis of SHPT. Administration of the FGF-23 antibody can markedly increase 1α-hydroxylase expression in kidney, which means that it can restore 1,25D levels significantly [27,28]. These findings suggested that the increase of FGF-23 may be the principal mechanism behind reduced 1,25D levels in early CKD.

**Figure 1:**
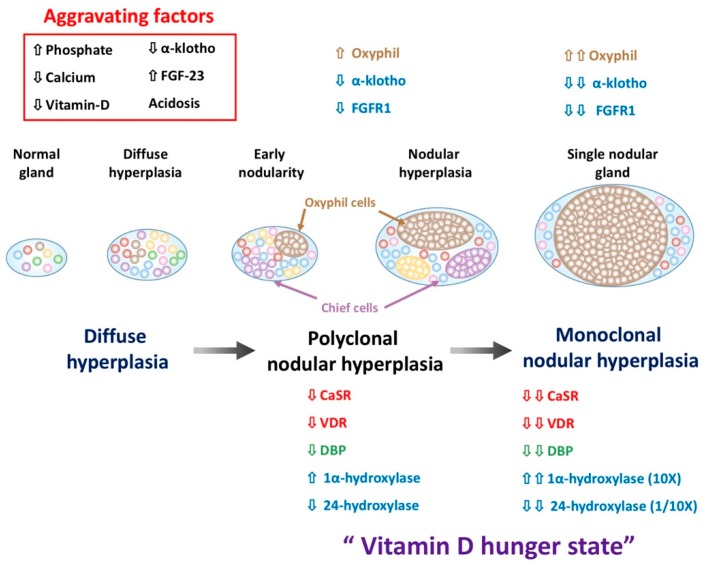
The development of parathyroid gland hyperplasia in secondary hyperparathyroidism (SHPT). In advanced SHPT, monoclonal cell growth vigorously that occupy the most of the gland and form a single large nodule. Both α-klotho and FGFR1 expression on parathyroid cells are decreased during the progress of hyperplasia and are negatively correlated with the volume of the hyperplastic parathyroid tissue. The reduced VDR and CaSR expression is prone to nodular hyperplasia and is considered to be in a relation to calcitriol or calcimimetics resistant. Increased 1α-hydroxylase and decreased 24-hydroxylase expression in secondary hyperplasia PTG cells would highlight the requirement of more 25D in SHPT. In parathyroid cell, the translocation of vitamin D from cytosol into mitochondria for 1,25D synthesis with the help of cytosolic DBP, and reducing the cytosolic DBP content within oxyphilic cell predominant parathyroid nodules might decrease the amount of local intracellular 1,25D production. This hydroxylase enzyme and cytosolic DBP change highlight the requirement of more 25D in SHPT, called vitamin D hunger. Increasing the serum level of 25D increases the intra-parathyroid free and bound 25D levels, which might overcome the decreased DBP levels, and improve the vitamin D hypo-responsiveness state in PTG among SHPT patients.(Abbreviation: SHPT, secondary hyperparathyroidism; FGFR1, fibroblast growth factor receptor 1; VDR, vitamin D receptor; CaSR, calcium sensing receptor; 1,25D, 1,25-dihydroxy vitamin D; PTH, parathyroid hormone; PTG, parathyroid gland; VDD, vitamin D deficiency, DBP: vitamin D binding protein; 25D, 25-hydroxy vitamin D.).

As hypocalcemia, 1,25D and 25-hydroxyvitmain D (25D) deficiency worsen in CKD progression, a general increase in the total number of parathyroid cells with a normal lobular structure occurs called diffuse hyperplasia. After progressing into the end stage of renal disease or even dialysis dependent status, SHPT becomes more severe and PTG becomes grossly enlarged and exhibits some nodular formation (nodular hyperplasia) (Figure 1). In advanced SHPT, the multi-nodule may develop into a single large nodule [29]. Once nodular hyperplasia in SHPT is established, these glands might be refractory to medical treatment and surgical parathyroidectomy is indicated [30]. Hyperphosphatemia is a main risk factor aggravating the severity of PTG hyperplasia and dialysis vintage and serum PTH level are also in a relation with nodular hyperplasia [31].

Pathophysiologically, hyperplasia precedes the decrease in CaSR expression. The decrease in vitamin D receptor (VDR) is parallel to the increases in hyperplastic growth and contributes to decrease the induction of the CaSR by VDRA [32,33]. Down regulation of CaSR may be attributed by parathyroid cell hyperplasia, but not uremia per se [33]. Inadequate CaSR and VDR density in PTG cause the poor response of extracellular calcium to suppress PTH and failure of calcitriol (1,25-dihydroxyvitamin D_3_) in treating SHPT. In general, parathyroid hyperplasia presented in CKD stage 5 patients with PTH > 400 ng/mL [34]. A PTG weight over 500 mg predicted nodular hyperplasia and this is equivalent to an estimated value of 330 mm^3^ [35]. In addition, a PTG volume > 300 mm^3^ or maximum diameter > 8 mm predicted nodular hyperplasia [36,37]. Furthermore, a PTG volume > 500 mm^3^ or maximum diameter > 10 mm might be refractory to the calcitriol treatment to SHPT.

In the kidney, 25D can be taken by the proximal renal tubule with the help of the cell surface receptors megalin and cubulin. The conversion of 25D into its active biological form, 1,25D, is in its majority catalyzed by mitochondrial 1α-hydroxylase and in the minority by microsomal enzymes [38,39]. 1,25D then binds to DBP then delivered to target tissue to regulate a wide variety of VDR genes.

1α-hydroxylase is a cytochrome P450 enzyme that in humans is encoded by the CYP27B1 gene [40]. 1α-hydroxylase is the key enzyme responsible for 1,25D biosynthesis. Activity of renal 1α-hydroxylase is the key that determines serum level of 1,25D. The 1α-hydroxylase gene is mainly expressed in the renal proximal tubule and is also expressed at lower levels in extrarenal sites such as PTG [41], osteoblasts in bones [42,43], vascular endothelial cells [44], the brain [45] and placenta [46,47]. 1,25D produced in the kidney exerts it endocrine effect to regulate bone and mineral hemostasis. Renal 1α-hydroxylase is tightly controlled by several physiological factors. For example, PTH [48], calcitonin [49] and hypophosphatemia [50,51] can promote 1α-hydroxylase gene expression, whereas FGF-23 and 1,25D suppress it. PTH and calcitonin positively induce renal CYP27B1 gene expression via stimulating cyclic AMP (cAMP) generation [52,53,54] and activating the cAMP-responsive elements (CRE) site within the gene promoter for renal 1α-hydroxylase (CYP27B1) [55], respectively. FGF-23 can both influence the synthesis of 1,25D in the proximal tubule of the kidney by the suppression expression of CYP27B1 and increase the activity of 24-hydroxylase [56,57]. Hypocalcemia can also enhance renal 1α-hydroxylase activity, but much of this effect is mediated indirectly through PTH. 1,25D inhibits the 1α-hydroxylase activity through inhibiting PTH, increasing FGF-23 production; and inducing the degradation enzyme 24-hydroxylase [49]. In the 24-hydroxylase knock out mice, serum 1,25D levels were inconclusively high indicating that the excessive 1,25D cannot suppress its own synthesis [3]. Therefore, 1,25D regulation on vitamin D metabolism is predominantly through enhancing 24-hydroxylase degradation rather than negative feedback on1α-hydroxylase activity.

24-hydrolyase is the enzyme responsible for the catabolism of 1,25D and ultimately leads to the excretion of the hormone as calcitroic acid [58]. In fact, both 25D and 1,25D are substrates for the hydroxylation reaction of 24-hydrolyase (CYP24A1). 24-hydrolyase is capable of the hydroxylation of the C23 or C24 side-chain carbons of 25D and 1,25D and results in the side chain cleavage and oxidation to carboxylic acid in C24 hydroxylation and lactone formation in C23 hydroxylation [59]. CYP24A1 is expressed in many, if not all, target cells containing VDR, including kidney, bone, intestine [60]. Both 1,25D and VDRA are potent inducer of 24-hydroxylase gene expression to avoid excess of circulating 1,25D and reduce risk of hypercalcemia. 1,25D is the preferred substrate relative to 25D [58], because the affinity of 24-hydroxylase for 1,25D is 25-times higher than that for 25D, which mean the 24-hydroxylase can safely and effectively prevent the accumulation of toxic levels of 1,25D after large dose of 25D supplement.

## 3. The Alteration of Vitamin D Metabolism in CKD

### 3.1. Decrease Vitamin D Synthesis and Increase Vitamin-D Catabolism in CKD

In CKD, PTH synthesis is increased in response to both 1,25D deficiency and hypocalcemia, then PTH stimulates renal CYP27B1 expression to rescue the 1,25D level. 1,25D consequently induces VDR-mediated intestinal calcium absorption to keep calcium homeostasis. PTH also downregulates renal CYP24A1 mRNA transcription, a 24-hydroxylase enzyme responsible for vitamin D degradation, and leads to attenuating 25D and 1,25D degradation via the cAMP/PKA signaling pathway [61,62].

As PTH controls blood calcium to keep serum calcium homeostasis, FGF-23 regulates the serum phosphate level and is involved in vitamin D metabolism. Hyperphosphatemia can induce osteocytes and osteoblasts to express FGF-23 and subsequently reduces phosphate reabsorption by inhibiting NaPi-IIa activity directly and indirectly by inhibiting renal CYP27B1 expression to lower blood 1,25D level, then reduces intestinal phosphate absorption [63]. Additionally, FGF-23 induces renal CYP24A1 expression to degrade 25D and 1,25D levels [62].

The function of PTH and FGF-23 in regulating CYP27B1 works in a reciprocal manner and compete with each other on CYP27B1 transcription. The direct administration of recombinant FGF-23 or its overexpression in mice induces a dose-dependent decrease in renal CYP27B1 mRNA expression, an increase in renal CYP24A1 mRNA expression, and a consequent decrease in serum 1,25D concentrations [28]. Instead, the administration of FGF-23 antibodies can increase renal CYP27B1 mRNA and decrease renal CYP24A1 mRNA to restore serum 1,25D concentration to normal. These changes are followed by increased serum calcium level, leading to decreased serum PTH [27]. Hence, FGF-23, rather than PTH, is a primary factor accounting for inappropriately low serum 1,25D concentration in CKD since the early stage of CKD. In brief, an increase of FGF-23 in CKD follows 1,25D deficiency and hypocalcemia, thereby increasing the PTH level and results in SHPT in CKD. The FGF-23 action may aggravate VDD if concurrently used with calcitriol or VDRA analogs during SHPT treatment as both FGF-23 and VDRA analogs both downregulate CYP27B1 and upregulate CYP24A1 expression to degrade 25D and 1,25D.

There is also another metabolic factor commonly presented in CKD that disturbs CYP27B1 expression such as diabetes [64], acidosis [65], and hyperuricemia [66,67]. Therefore, high FGF-23 and CKD related metabolic factors are associated with CYP27B1 transcription inhibition in CKD.

Lower 25D bioavailability in CKD is also another cause of VDD. As limited sun exposure and dietary vitamin D intake, less 25-hydroxyvitmain D filtered by declining GFR, diminished megalin expression, and albuminuria increase filtered 25-hydroxyvitmain D lost in urine are all aggravating factors that lead to 25D shortage and cannot provide an inadequate substrate for 1α-hydroxylase and worsens VDD in CKD [46,68].

### 3.2. Nutritional Vitamin D Hunger in the PTG

In normal physiological conditions, FGF-23 can directly suppress PTH production by directly inhibiting PTH transcription and secretion, and indirectly by increasing parathyroid 1α-hydroxylase activity [69]. FGF-23 can also increase CaSR and VDR expression and decrease PTG volume. However, low PTG α-Klotho and FGFR1 expression lets FGF-23 lose its inhibitory effect on parathyroid cells and fails to increase CaSR and VDR [70]. Moreover, the administration of FGF-23 in CKD animals cannot reduce the PTH level, which indicates FGF-23 resistance in PTG caused by the low expression of α-Klotho and FGFR1 [71]. In summary, in patients with CKD, FGF-23 levels increase progressively to compensate phosphate retention, but the high FGF-23 levels fail to suppress PTH secretion due to decreased Klotho-FGFR1 complex expression in hyperplastic PTG, called FGF-23 resistance. Furthermore, recent literature in dialysis patients of SHPT has shown that the expression of α-Klotho and FGFR1 is decreased in PTG of dialysis patients and were negatively correlated with the volume of the hyperplastic parathyroid tissue [72].

Compared with the normal gland, the mRNA expression and protein level for 1α-hydroxylase (CYP27B1) in secondary hyperplastic parathyroid cells is higher [73]. Increased 1α-hydroxylase (approximately 10-fold) and decreased 24-hydroxylase (approximately 1/10-fold) concentrations are found in 78% of secondary hyperplasia PTG cells and highlight the requirement of more 25D in SHPT [74]. The expression of 1α-hydroxylase is much higher in oxyphil cells than chief cells, which is the dominant cell group in SHPT. Calcimimetics treatment had a further 42% increase in parathyroid 1α-hydroxylase mRNA and 2.2-fold decrease in 24-hydroxylase mRNA that resulted in an ~53% decrease in PTH mRNA [75]. Besides, the decrease of megalin expression in the parathyroid gland may decrease 25D uptake and mediate the demand for more circulating 25D to correct PTH synthesis. Hence, the requirement for a substrate for vitamin D synthesis dramatically increases in SHPT and becomes hungrier if receiving treatment of calcimimetics in severe SHPT, called “vitamin D hunger status” as SHPT progresses in CKD. Therefore, more evidence in the data have overwhelmingly indicated the adjuvant role of NVD in SHPT prevention and PTH lowering effect in combination with calcitriol or calcimimetics treatment.

## 4. The Change of Parathyroid Gland Cell Membrane CaSR and Nuclear VDR Expression in SHPT

As SHPT progresses, the expression of VDR and CaSR are both reduced in PTG hyperplasia and the CaSR becomes more decreased if PTG has nodular progression (Figure 1) [76]. Decreasing these two receptors would limit the inhibitory ability of calcium and 1,25D in PTH secretion and also hinder the therapeutic effect of calcimimetics or calcitriol. Thus, the way to increase VDR and CaSR expression in parathyroid nodular hyperplasia is reasonable to improve the efficiency of calcimimetics or calcitriol.

## 5. The Change of Parathyroid Cells Composition in SHPT

### 5.1. Increase Oxyphil Cells Proporation in PTG of SHPT

The PTG is composed of two cell types: chief cells and oxyphil cells. Chief cells are the predominant cell in PTG and are responsible for PTH production and secretion in response to low extracellular calcium levels, sensed by CaSR on the plasma membrane of chief cells. The oxyphil cell form clusters that are scattered between the chief cell and their function is still unknown. Histologically, oxyphil cells are larger than the chief cells and its cytoplasm is more eosinophilic due to higher mitochondrial content. Oxyphil cells have been thought to be derived from chief cells as both chief cells and oxyphil cells express PTH and glial cells missing 2 (GCM2), a parathyroid-specific transcription factor that is essential for parathyroid cell development [77,78]. Another possible explanation of oxyphil cell transdifferentiation was proposed by Müller-Höcker et al. where the defective mitochondrial respiratory chain in chief cells would induce mitochondrial mutation, consequently, mitochondrial proliferation can compensate it and transdifferentiate into oxyphil cells [77].

In young healthy adults, chief cells are dominant cells in PTG and oxyphil cells only account for 1% of PTG cells [79,80]. However, chief cells are gradually replaced by oxyphil cells as they increase with age. Oxyphil cells dramatically increase in the number in hyperplastic PTG and are considered to function to synthesize and secrete PTH, which contribute to the pathophysiology of SHPT due to the heterotransplantation of the human parathyroid nodule that is exclusively composed of chief cells or oxyphil cells into nude mice that are capable of secreting intact human PTH [81]. As with chief cells, oxyphil cells can also synthesize calcitriol to suppress parathyroid proliferation [82], enhance PTH secretion in response to a hypocalcemic stimulus, and even secrete PTH related protein (PTHrP) [83,84].

### 5.2. The Alteration of Parathyroid Cell Composition after SHPT Treatment

The proportion of oxyphil cells was significantly increased in CKD and had a positive correlation with total PTG weight in SHPT, which suggests that oxyphil cell proliferation is sensitive to PTG stimulation [85]. After calcimimetics treatment, the oxyphil cell content was significantly higher than in the paricalcitol group (a less calcemic analog of calcitriol) [80,86]. In addition, in a retrospective analysis, the proliferation of oxyphil cells was the major pathological progression in SHPT and was highly associated with calcitriol treatment. The dose and duration of oral calcitriol treatment was an independent predictor of the high oxyphil cell ratio [87]. These findings underline that higher oxyphil cell proportion among PTG cells in treatment resistance is associated with the calcemic effect on CaSR activation, following the chief-to-oxyphil cell transdifferentiation. The demand of NDV is dramatically raised in severe or refractory SHPT as the expression of 1α-hydroxylase is parallel to the number of oxyphil cells in PTG. Thus, the adequate supplement of NVD might be good for PTH lowering therapeutic effects. Our previous study also revealed that using NVD on a combination of VDRA analogs and low dose calcimimetics may provide a beneficial effect in patients with severe or refractory SHPT [17].

### 5.3. The Regression of Parathyroid Hyperplasia after SHPT Treatment

**Figure 2:**
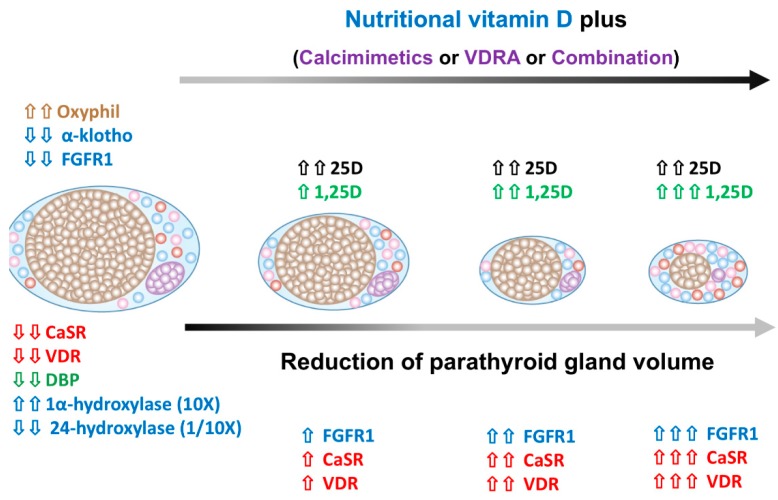
Parathyroid gland regression after parathyroid hormone (PTH) lowering therapy. Calcitriol and calcimimetics can effectively reduce PTG volume in SHPT and concurrently increase VDR and CaSR expression to improve the efficient SHPT treatment. NVD supplement meets the demand of parathyroid 25D requirement and lower PTH by dramatically increasing intra-gland 1,25D production. (Abbreviation: PTG, parathyroid gland; SHPT, secondary hyperparathyroidism; VDR, vitamin D receptor; CaSR, calcium sensing receptor; NVD, nutritional vitamin D; 25D, 25-hydroxy vitamin D; PTH, parathyroid hormone; 1,25D, 1,25-dihydroxy vitamin D.).

Calcitriol can effectively lower the PTH level in hemodialysis patients with a concomitant reduction in PTG volume after 12 weeks of treatment (Figure 2) [88]. Fukagawa et al. proposed that an initial PTG size > 0.5 cm^3^ was highly associated with calcitriol resistance [89]. This finding was further supported by histological analysis after parathyroidectomy, as PTG size > 0.5 cm^3^ may exhibit nodular formation [35].

Calcimimetics appear to be a safe and effective therapy to lower serum calcium, phosphorus, PTH level, and attenuated PTG hyperplasia in CKD animals [90,91]. Calcimimetics increased both CaSR and VDR expression in PTG and facilitated the inhibitory effect of calcitriol or VDRA analogs on PTH synthesis and parathyroid cell proliferation, and thereby resulted in PTG hyperplasia regression. The precise mechanism in reducing the PTG size after calcimimetics treatment remains a matter of discussion and a decrease in parathyroid cell volume [92] or cell number decrease, or parathyroid cell apoptosis [79,93] have all been proposed.

Calcimimetics directly elicit a rapid increase of CaSR and VDR mRNA in uremic rat PTG within two weeks. Calcitriol also increases CaSR mRNA, but seems more related to the control down of parathyroid hyperplasia than acting on VDR [94]. Actually, calcimimetics upregulate CaSR earlier than calcitriol as calcitriol affects cell proliferation through nuclear receptor transcription action, but calcimimetics sensitize hyperplastic PTG to the extracellular calcium level by the increased expression of CaSR. Treatment of cinacalcet for four weeks can effectively prevent the increase in parathyroid cell proliferation [94]. These findings highlight the synergistic effect of calcimimetics and calcitriol in the treatment of SHPT, and calcitriol has an add on effect on CaSR and VDR expression with calcimimetics.

## 6. Vitamin D Binding Protein (DBP)

### 6.1. Role of DBP in Parathyroid 1,25D Synthesis

In general, vitamin D circulating in blood is composed of three forms: (1) 85–90% are bound to DBP, a specific transport protein for vitamin D; (2) 10–15% are found in serum albumin with lower affinity; and (3) only a tiny amount (less than 1%) in a free unbound form [95]. The sum of DBP-, albumin-bound, and free form of vitamin D is referred to as the total vitamin D. 25D concentration in blood is nearly 1000 times greater than that of 1,25D. DBP has the highest affinity with 25D, but only a 10–100 folds lower affinity to 1,25D because of the steric hindrance of the additional hydroxyl group [96]. Because of relative weak affinity between 25D and albumin, the combination of free and albumin-bound 25D are often referred to as “bioavailable” vitamin D [97]. Whether free, bioavailable, or total 25D represents the biological activity of vitamin D in target cells is still debatable. The free hormone hypothesis states that only hormones free from binding protein are capable of moving lipophilic steroid hormones through the cell membrane to bind to their intracellular receptors and exert its biological function [98]. In a large prospective cohort study of incident hemodialysis patients, Bhan et al. reported bioavailable, but not total 25D and 1,25D levels were significantly correlated with serum calcium level. Additionally, bioavailable 25D was significantly associated with PTH level [97]. Furthermore, free and bioavailable 25D are more strongly correlated with bone mineral density than total 25D in health young adults [98].

In brief, the free D hypothesis states that vitamin D hydroxyl metabolites in the circulation are primarily bound to DBP and to a lesser extent, to albumin and possibly to lipoproteins; only the unbound 25D and 1,25D freely crossing the cell membrane through the nuclear pore to interact with the VDR [99]. Many tissues express the 1α-hydroxylase that metabolizes 25D to the active 1,25D locally. In several tissues, such as the kidneys, parathyroid glands and placenta, 25D and 1,25D bound to DBP may enter cell tissues by endocytosis via megalin/cubulin and are not limited to diffusion by the free hormones [100].

Plasma DBP level is not decreased or even higher in CKD patients despite the urinary loss of DBP, indicating the compensatory enhanced production of DBP. Thus, the plasma DBP level is not an appropriate predictor of vitamin D deficiency in CKD patients [101]. In a previous proteomic study, intracellular DBP was found to be significantly down-regulated in parathyroid gland cell, especially among oxyphilic cell predominant parathyroid nodules, and this was assumed to be closely associated with calcitriol resistance in SHPT patients [84].

Our previous studies demonstrated that NVD had a positive benefit as an adjuvant therapy with paricalcitol/calcitriol and calcimimetics in treating SHPT in dialysis patients [16,17]. These clinical studies also suggested that increase serum level of 25D may contribute to the more adequate control of SHPT. Thus, we speculated that, for parathyroid cell, the translocation of vitamin D from cytosol into mitochondria for 1,25D synthesis with the help of cytosolic DBP, and reducing the DBP content within the cell might decrease the amount of local intracellular 1,25D production. Increasing the serum level of 25D increases the intra-parathyroid free and bound 25D levels, which might overcome the decreased DBP levels, and improve the vitamin D hypo-responsiveness state in PTG among SHPT patients.

### 6.2. Do DBP Genotypes Influence Intrinsic 1,25D Production in the Parathyroid Gland?

Variation in the DBP gene coding region significantly produces proteins with different serum concentration and affinity to different vitamin D metabolites. Three common allelic forms of DBP, known as group-specific component (Gc) 1F, 1S, and 2, are presented in serum at different concentrations (1F > 1S > 2) and exhibit varying affinities for 25D and 1,25D (1F > 1S > 2). Different forms of DBP influence the transport of vitamin D metabolites to peripheral target cells, for example, the DBP genotype determines the bioavailability of 25D into cultured monocytes [102] and keratinocytes [103]. This suggests that monocytes respond to free 25D rather than DBP-bound 25D. The antibacterial response (cathelicidin production) is more dominant in DPB Gc 1S and Gc 2 [104]. In addition, the DBP genotype also influences serum 25D and 1,25D levels in circulation, thus the threshold for VDD may differ between Gc genotypes [105,106]. The DBP genotype also influences renal 1,25D synthesis by facilitating 25D reabsorption into the proximal renal tubule, and this effect appears to be more related to DBP Gc 1F [105]. The function of DBP is not merely to act as a serum reservoir, but also as a carrier protein to the target cell, like the thyroid hormone-binding globulin or sex hormone-binding globulin. DBP can prolong the circulating half-life of vitamin D and also regulate its immediate bioavailability to target tissue. Currently, there is no available literature discussing the effects of parathyroid DBP levels and their genotypes on 25D transportation within PTG. Future studies need to explore the DBP levels among different genotypes, mechanisms regarding vitamin D transport and metabolism within PTG and different treatment responses in SHPT patients.

## 7. The Negative Control of 1,25D on Vitamin D Metabolism

Although a number of tissues have been demonstrated to express 1α-hydroxylase, the expression of that in kidney is the main source of circulating 1,25D. Renal 1α-hydroxylase is tightly regulated by three hormones: PTH, FGF-23 and 1,25D itself [107]. PTH stimulate 1α-hydroxylase but FGF-23 and 1,25D inhibit it. The mechanism of 1,25D inhibits 1α-hydroxylase activity is through inhibiting PTH and increasing FGF-23 production and inducing the degradation enzyme 24-hydroxylase [49]. Besides, 1,25D can also directly inhibit CYP24A1 expression by VDR and vitamin D inhibitory receptor (VDIR) and diminish its transcription [108]. The difference in the serum levels of 1,25D upon i.v. administration of 1-hydroxyvitamin D is due to the continuous conversion of the 1-hydroxyvitaminD to 1,25D by the hepatic 25-hydroxylase, while the half-life of i.v. calcitriol is of a few hours due to its degradation by 24-hydroxylase. In the 24-hydroxylase knock out mouse, serum 1,25D levels are very high conclusively indicating that the excessive 1,25D cannot suppress its own synthesis [109]. Hence, 1,25D affect vitamin D metabolism is predominantly regulated by enhancing 24-hydroxylase degradation rather than negative feedback of 1α-hydroxylase activity.

## 8. Synergistic Action of 25D and 1,25D on PTH Suppression

The relative potencies of 25D and 1,25D in reducing PTH secretion have been demonstrated in primary cultures of bovine parathyroid cells (bPTC) [41]. Physiological concentration of 25D has inhibitory effects on PTH transcription and thought to be related to endogenous 1α-hydroxylase activity within the parathyroid cells [110]. 25D is several hundred times less potent than 1,25D in suppressing PTH secretion and transcription in bPTC due to its lower affinity to VDR [41]. ADAM17 and transforming growth factor-α (TGF-α) synergy reinforce the vicious cycle of epidermal growth factor (EGF) receptor activation which is related to PTG growth. 1,25D induce C/EBPβ gene expression to suppress ADAM17 promoter activity and then suppress PTG growth. In SHPT, 25D been proved to have synergistic effect with 1,25D on PTG growth suppression [111]. Nevertheless, this synergistic effect is conclusively demonstrated in vitro and need further animal and clinical exploration.

## 9. The Role of Vitamin-D Deficiency (VDD) on PTH Metabolism

CKD has an impact on PTH metabolism at least in part through downregulation of VDR, competitive inhibition between (1–84) PTH and its fragments and PTHR1 dysfunction and inhibitory or competing downstream signal [107]. Previous experimental studies revealed that circulating uremic toxins may trigger cellular oxidative stress [112]. Subsequent studies showed indoxyl sulfate and inflammatory bioactive lipids (e.g., ox-LDL), as the most likely culprit [113,114]. Increased oxidative stress may be in the causal effect between CKD and PTH hyporesponsivenes. Our previous study showed T cell differentiation correlated with serum 25D level in HD patients. Low serum 25D level contributes to the development of inflammation and oxidative stress through decrease T helper 2 (Th2) differentiation [115]. Thus, avoid the VDD may attenuate the oxidative stress resulting in decreasing PTH hyporesponsiveness. This may contribute to decrease the possibility of increase PTH levels. Its clinical relevance, however, should be formally evaluated. In addition, supraphysiological dose calcitriol widely used in treating SHPT may disturbed bone remodeling process and further influence vascular calcification. Combined use of NVD in SHPT can attenuate the dose of calcitriol with comparable PTH-lowering effect [116].

## 10. Nutritional Vitamin D Prevents Developing SHPT in Early CKD 

VDD is highly prevalent in the CKD population. In a cross-sectional analysis of 825 consecutive patients new to HD, 78% were considered VDD (<30 ng/mL) and around 18% had severe deficiency (<10 ng/mL) and were associated with increased early mortality [117]. VDD is associated with elevated PTH levels and worsening the SHPT. Some studies have reported the high association between free 25D and serum PTH decline [92], bone turnover markers and bone mineral density [96,118], but others who have not reached such conclusions may be due to whether the total or free 25D represents the surrogate marker of vitamin D bioactivity [119,120]. NVD, both the cholecalciferol and calcifediol supplements are effective in increasing the total and free 25D level and are associated with serum PTH level decline [121]. In CKD patients, supplementing with cholecalciferol for 12 weeks had a significant increase (185%) in serum 25D concentration when compared with the placebo. Meanwhile, the PTH levels also tended to decrease after cholecalciferol supplementation [122]. More recently, Westerberg et al. reported high-dose cholecalciferol (8000 IU/day) in patients with CKD stage 3–4 halted the development of SHPT and did not increase the risk of hypercalcemia and hyperphosphatemia [123]. In non-dialysis CKD patients with VDD, cholecalciferol supplementation was superior to ergocalciferol with a two-fold increase of circulating 25D level [124]. The 2017 KDIGO CKD-MBD Guideline suggests that VDD should be corrected if CKD stage 3 to 5a not yet dialysis patients have a progressive or persistently high PTH level [3]. NVD supplements might be considered as the adjuvant therapy for SHPT prevention in not yet HD patients for the following reasons: (1) High prevalence of VDD in CKD patients; (2) NVD is the substrate for 1α-hydroxylase, whose activity is increased in SHPT; (3) 25D directly interacts with VDR in PTG; (4) NVD is relatively inexpensive and has a lower risk of toxicity, hypercalcemia, and hyperphosphatemia; and (5) NVD had a pleiotropic effect in our previous reports [46]. Although more evidences support the benefit of initiating NVD treatment if concurrently having VDD to lower the occurrence of SHPT, the efficiency of NVD supplement needs more randomized controlled trials to prove.

## 11. The Role of NVD in the PTH-Lowering Effect in Dialysis Patients Remains Controversial

The PTH lowering effect of NVD in dialysis patients is still inconclusive. Some studies have reported that NVD supplementation in VDD dialysis patients could significantly restore 25D levels, but the PTH lowering effect had a nonsignificant downward trend or did not reach statistical significance [125,126,127]. Kandula et al. in 2011 conducted a systemic review and meta-analysis that analyzed 17 observational cohort studies and five randomized controlled trials to compare the benefit and harm of vitamin D supplements in CKD with or without dialysis [128]. They reported a statistical improvement in serum 25D (mean difference 24.1 ng/mL, 95% CI 19.6 to 28.6) and an associated decline in PTH among the observational studies (mean difference −41.7 pg/mL, 95% CI −55.8 to −27.7). The PTH lowering effect is more prominent in dialysis patients than in non-dialysis patients. In another randomized controlled trial, there was a significant increase in serum 25(OH)D levels (mean difference 14 ng/mL, 95% CI 5.6 to 22.4) and an associated decrease in PTH levels (mean difference −31.5 pg/mL, 95% CI −57 to −6.1) with a low incidence of hypercalcemia (up to 3%) and hyperphosphatemia (up to 7%) after NVD supplementation [129]. The Dialysis Infection and Vitamin D in New England (DIVINE) was a randomized clinical trial (RCTs) study conducted in 2009 that evaluated the effect of high dose ergocalciferol (50,000 IU/week), low-dose ergocalciferol (50,000 IU/mo), or a placebo for 12 weeks on serum 25D, 1,25D, calcium, phosphate, and PTH levels. However, the results shown in 2015 revealed that oral ergocalciferol could increase 25D levels in maintenance HD patients, but did not have significant alterations in the calcium, phosphate, nor PTH levels in the 12-week follow-up [130].

## 12. NVD Has Adjuvant Benefits in Treating SHPT in Dialysis Patients

**Figure 3:**
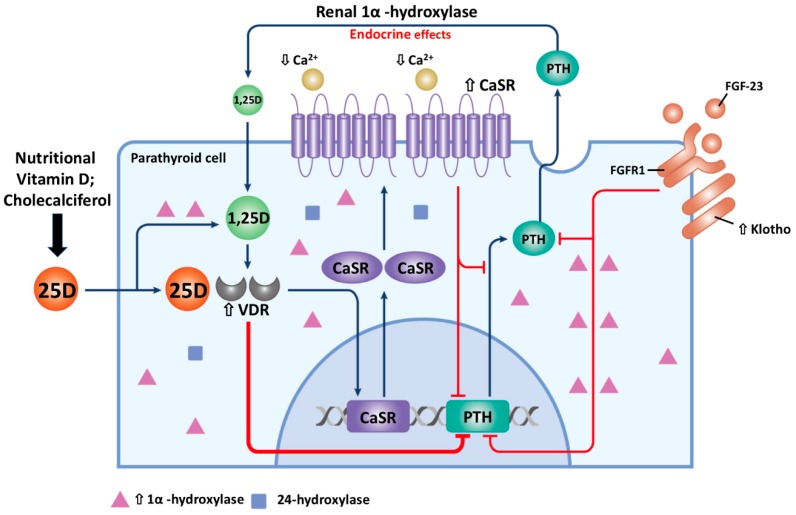
The adjuvant role of nutritional vitamin D (NVD) in SHPT treatment. NVD supplement had the benefit of increasing parathyroid 1,25D production and may synergize with calcimimetics and/or calcitriol by increasing CaSR and VDR expression which all contribute to suppress of PTH synthesis in PTG of SHPT. 1-hydroxylase and 24-hydroxylase are mitochondrial enzymes. (Abbreviation: NVD, nutritional vitamin D; 1,25D, 1,25-dihydroxy vitamin D; CaSR, calcium sensing receptor; VDR, vitamin D receptor; PTH, parathyroid hormone; SHPT, secondary hyperparathyroidism.).

NVD has additive benefits in treating SHPT if combined with calcitriol or calcimimetics in dialysis patients. We previously reported cholecalciferol in combination with paricalcitol for 15 weeks could effectively lower the PTH level in dialysis patients. Furthermore, a dose of 5000 IU/week of cholecalciferol could maintain a serum 25D concentration > 30 ng/mL as early as after eight weeks of supplementation [16]. In addition, we also found that cholecalciferol supplementation could additively reduce serum PTH levels, improve 25D levels, and improve femoral neck bone mineral density (BMD) when together with a calcimimetics and calcitriol combination in severe SHPT (PTH > 1000 pg/mL) dialysis patients [17]. Taken together, NVD had a positive effect as an adjuvant therapy with calcitriol and calcimimetics in treating SHPT in dialysis patients (Figure 3) as (1) NVD is a substrate for 1α-hydroxylase, whose activity is elicited in SHPT progression and further enhanced directly by the treatment of calcimimetics; (2) Calcimimetics promote the transdifferentiation of chief cells to oxyphil cells, and oxyphil cells are abundant in 1α-hydroxylase and CaSR; (3) Calcimimetics increase CaSR and VDR expression in PTG; and (4) Intra-gland 1,25D production can further suppress PTH by more VDR expression i01230525n PTG.

## 13. Conclusions

For the high prevalence of VDD in hemodialysis patients, an increase in 1α-hydroxylase to 10-fold and decrease in 24-hydroxylase to 1/10-fold highlight the requirement of more 25D in the PTG of SHPT. The expression of CaSR and VDR were also decreased in the PTG cells, which is thought to be related to calcimimetics or calcitriol resistance. A higher proportion of oxyphil cells as hyperplastic parathyroid progression, lower cytosolic DBP content in oxyphil cells, and calcitriol promote vitamin D degradation by enhancing 24-hydroxylase activity all aggravate vitamin D hunger in PTG. Hence, NVD supplementation in SHPT is relative important. Clinically, NVD supplements can effectively restore serum 25D concentration, alleviate the development of SHPT in early CKD, and have benefits in further lowering PTH in moderate to severe SHPT in dialysis patients. However, these benefit of NVD supplement in SHPT need more randomized control trials to prove.

## Abbreviation

25D	25-hydroxy vitamin D
1,25D	1,25-dihydroxy vitamin D
CKD	chronic kidney disease
CKD-MBD	chronic kidney disease-mineral bone disorders
CaSR	calcium sensing receptor
DBP	vitamin D binding protein
FGF-23	fibroblast growth factor-23
FGFR1	fibroblast growth factor receptor 1
HD	hemodialysis
PTH	parathyroid hormone
NVD	nutritional vitamin D
PTG	parathyroid gland
SHPT	secondary hyperparathyroidism
VDD	vitamin D deficiency
VDR	vitamin D receptor
VDRA	vitamin D receptor activator
